# Disulfide-Crosslinked Polyurethane-Modified Asphalt: Balancing Fatigue Resistance and Healing Through Dynamic Covalent Networks

**DOI:** 10.3390/polym18050582

**Published:** 2026-02-27

**Authors:** Yemao Zhang, Xijuan Zhao

**Affiliations:** 1 Nanjing Institute of Technology, Nanjing 211167, China; 2 Jiangsu Key Laboratory of Intelligent Construction and Smart Operation & Maintenance of Power Infrastructure, Nanjing Institute of Technology, Nanjing 211167, China

**Keywords:** thermosetting polyurethane, modified asphalt, self-healing, fatigue, disulfide crosslinking

## Abstract

Thermosetting polyurethane (PU) has recently been introduced as an asphalt modifier to improve the mechanical strength and durability of pavements. However, the permanent crosslinked network of thermosetting PU makes the material difficult to repair once damage accumulates. In contrast, self-healing asphalt technologies rely on either extrinsic healing agents or intrinsic dynamic bonds to restore stiffness and delay cracking. Dynamic disulfide bonds are a promising class of reversible covalent bonds that can rearrange at moderate temperatures and have been widely used to build self-healing polyurethane networks. This study investigates a disulfide-crosslinked polyurethane-modified asphalt binder (DP10) and compares its fatigue and healing performance with base asphalt (BA), thermosetting PU-modified asphalt (P10), and styrene–butadiene–styrene (SBS)-modified asphalts (S3 and S10). A dynamic shear rheometer (DSR) was used to conduct time sweep fatigue tests, linear amplitude sweep (LAS) tests, and fatigue–healing–fatigue protocols. Fourier transform infrared spectroscopy (FTIR) was employed to confirm the formation of polyurethane and disulfide structures. Results show that DP10 significantly increases fatigue life at small to medium strain levels compared with BA and P10 and performs competitively with SBS-modified binders. More importantly, DP10 exhibits a much higher healing index than P10 and maintains strong healing capability over repeated fatigue–healing cycles, approaching the intrinsic healing level of base asphalt. These findings demonstrate that incorporating dynamic disulfide bonds into thermosetting PU networks provides a practical route to binders that combine high strength with recoverability, which is attractive for long-life, self-healing pavement design.

## 1. Introduction

Asphalt binders are widely modified with polymers to improve rutting resistance, cracking resistance, and long-term durability of pavements. Conventional elastomeric modifiers such as styrene–butadiene–styrene (SBS) can enhance high-temperature stiffness and fatigue resistance, but their performance is still limited under severe loading and environmental conditions, and their phase-separated morphology can compromise storage stability and aging resistance [1,2]. In recent years, thermosetting polyurethane (PU) has attracted growing attention as a reactive asphalt modifier. Due to its continuous crosslinked network and strong chemical bonding with bitumen components, thermosetting PU-modified asphalt binders and mixtures often show excellent high-temperature deformation resistance, tensile strength, low-temperature toughness, and moisture damage resistance [3,4].

Despite these advantages, thermosetting PU systems suffer from an inherent drawback: once the three-dimensional network is formed and microcracks initiate, the damage is essentially irreversible [5,6,7]. The dense crosslinking that gives high stiffness and strength also suppresses chain mobility and limits the ability of the material to relax stresses or close cracks. Under repeated loading, this can lead to rapid accumulation of fatigue damage and loss of serviceability, even if the initial performance is superior to that of SBS-modified binders. For long-life pavements, it is therefore desirable to design PU-based modifiers that not only strengthen the binder but also allow partial recovery of stiffness and damage under realistic thermal conditions [8,9,10].

Self-healing asphalt has emerged as a promising concept to address fatigue and microcracking in pavements. The healing mechanisms can be broadly classified into extrinsic and intrinsic approaches. Extrinsic self-healing relies on externally supplied healing agents, commonly encapsulated in microcapsules or stored in vascular networks. When cracks form, the capsules or channels rupture and release low-viscosity agents that flow into the damaged region and rebond the matrix [11,12]. In asphalt, numerous studies have proposed microcapsules containing rejuvenators or reactive monomers, as well as induction heating or microwave heating to stimulate healing. While these methods can be effective, they depend on a finite reservoir of healing agent, and the healing efficiency tends to decrease once the capsules are depleted or the conductive additives are degraded [13,14,15].

Intrinsic self-healing, in contrast, is achieved by introducing reversible interactions into the polymer network itself. These can be dynamic non-covalent interactions such as hydrogen bonding, π–π stacking, ionic interactions, or metal–ligand coordination, or dynamic covalent bonds such as Diels–Alder adducts, imine bonds, disulfide bonds, and others. In such systems, the network can rearrange under appropriate stimuli (temperature, light, pH, etc.), allowing broken bonds to reform and the material to recover mechanical properties multiple times [16,17]. For polyurethane networks, dynamic covalent chemistry has been widely used to design self-healing elastomers that respond to modest heating, showing high healing efficiencies and good mechanical robustness.

Among different dynamic covalent bonds, disulfide bonds (S–S) are particularly attractive. Disulfide bonds can undergo reversible exchange reactions through homolytic or nucleophilic mechanisms, and their activation temperature can be tailored by adjusting the local chemical environment [18,19]. Disulfide-containing polyurethane networks have been shown to combine good tensile properties with high self-healing efficiency at relatively mild temperatures, due to the synergistic effect of covalent disulfide metathesis and non-covalent hydrogen bonding. Recent work has extended this concept to asphalt systems by synthesizing disulfide-bearing polyurethane elastomers and blending them with bitumen. These studies reported that the introduction of disulfide bonds into the PU backbone significantly enhances the healing efficiency at the binder–elastomer interface and improves crack closure in the asphalt binder. Dynamic covalent networks based on other chalcogen bonds (e.g., diselenide) have also been shown to impart strong self-healing capability to asphalt binders [20,21].

However, most existing work on self-healing polyurethane-modified asphalt has focused either on thermoplastic PU or on relatively soft elastomer phases, and there is still limited understanding of how dynamic disulfide bonds can be used to “soften” the otherwise rigid network of thermosetting PU-modified asphalt [22,23]. In particular, there is a need to quantify how disulfide crosslinks affect: (1) the fatigue resistance under different strain levels, (2) the healing efficiency over repeated damage–healing cycles, and (3) the balance between stiffness and recoverability compared with conventional SBS-modified binders. At the same time, test methods such as time sweep (TS) and linear amplitude sweep (LAS) in a dynamic shear rheometer (DSR) have become standard tools for characterizing binder-level fatigue behavior and can provide a consistent framework to evaluate dynamic PU systems [24,25].

In this study, a thermosetting polyurethane-modified asphalt binder (P10) and a disulfide-crosslinked polyurethane-modified asphalt binder (DP10) were prepared by reacting a polyether polyol with an isocyanate in the presence or absence of a disulfide-containing chain extender. Their performance was compared with a base asphalt (BA) and two SBS-modified binders with different polymer contents (S3 and S10). Fourier transform infrared spectroscopy (FTIR) was used to confirm the formation of urethane and disulfide structures. Time sweep fatigue tests, LAS tests, and fatigue–healing–fatigue protocols were carried out in a DSR to assess fatigue life, damage evolution, and healing indices at binder level. The original contributions of this study can be summarized as below: (1) introducing a dynamic disulfide-containing chain extender (HEDS) into an in situ formed thermosetting PU network in asphalt to reconcile stiffness and healing; (2) benchmarking against conventional SBS-modified binders at both normal (3 wt%) and high (10 wt%) dosages; and (3) demonstrating repeatable healing performance through multiple fatigue–healing–fatigue (F–H–F) cycles, beyond single-cycle recovery tests.

## 2. Materials and Methods

**Figure 1:**
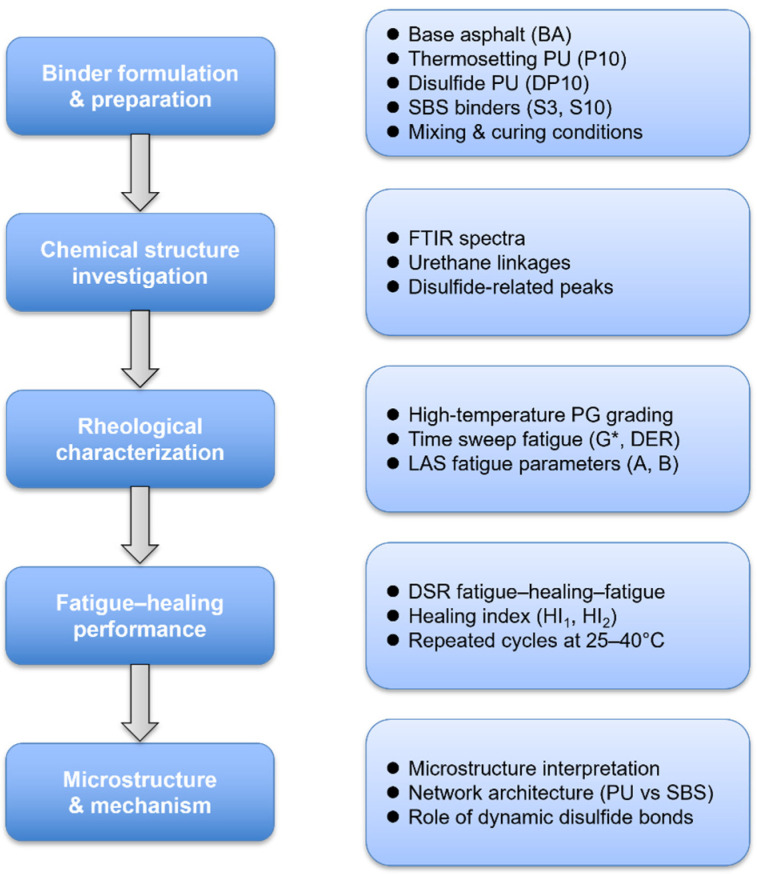
Flowchart of experimental design in this study.

The overall experimental design of this study was presented in Figure 1.

### 2.1. Materials

A pen #70 grade base asphalt (BA) was selected as the reference binder. Its basic physical properties, including penetration at 25 °C, softening point, ductility, and rutting factor at the design high temperature, were determined according to standard test methods, as summarized in Table 1.

A two-component polyurethane system was used to prepare the thermosetting and disulfide-crosslinked PU networks. The polyol component (A) was a poly(oxypropylene) diol (PPG, purchased from Thermo Fisher Scientific, Waltham, MA, USA) with an appropriate hydroxyl value to react with the isocyanate. The isocyanate component (B) was 4,4′-diphenylmethane diisocyanate (MDI, purchased from Thermo Fisher Scientific, Waltham, MA, USA). A disulfide-containing diol, 2-hydroxyethyl disulfide (HEDS, purity 98%, purchased from Tokyo Chemical Industry, Tokyo, Japan), was used as a chain extender to introduce dynamic disulfide bonds into the PU network.

For comparison, styrene–butadiene–styrene (SBS) block copolymer was used to produce conventional polymer modified binders. Two SBS-modified binders with polymer contents of 3 wt% and 10 wt% were prepared and are denoted as S3 and S10, respectively. The binders discussed throughout the paper are summarized in Table 2.

### 2.2. Preparation of Modified Binders

#### 2.2.1. Thermosetting PU-Modified Asphalt (P10)

The thermosetting PU-modified asphalt (P10) was prepared by in situ polymerization in the asphalt matrix. First, the base asphalt and the polyol component (PPG) were mixed to form component I. This blend was heated to approximately 100 °C under stirring to ensure homogeneity. In parallel, the isocyanate component (MDI) was preheated to about 60 °C to reduce viscosity.

The two components were then combined: component I and MDI were mixed at a controlled speed (about 500 rpm) for 2–3 min to initiate the urethane-forming reaction. The total PU content in the asphalt binder was fixed at 10 wt% with respect to the mass of asphalt. After mixing, the system was allowed to cure at 100–120 °C to complete network formation. The curing temperature and time were selected to promote full reaction of the isocyanate groups and to form a continuous thermosetting PU network within the bitumen.

#### 2.2.2. Disulfide-Crosslinked PU-Modified Asphalt (DP10)

The disulfide-crosslinked PU-modified asphalt (DP10) was prepared following the same procedure as for P10, with the addition of the disulfide-containing chain extender. Specifically, 2-hydroxyethyl disulfide (HEDS) was added to component I (base asphalt + polyol) before mixing with MDI. In DP10, HEDS partially replaced PPG in the polyol component at 50 mol% on a diol (–OH equivalent) basis. The overall NCO/OH equivalent ratio was kept at 1.05, consistent with P10. For a representative batch containing 100 g base asphalt, the total PU precursor dosage was fixed at 10 g (10 wt% relative to asphalt), consisting of 7.463 g PPG, 0.576 g HEDS, and 1.961 g MDI. After addition of HEDS, the mixture was heated to 100 °C and stirred until homogeneous. The preheated MDI was then added, and the system was mixed at 500 rpm for 2–3 min. The binder was subsequently cured at 100–120 °C, similar to P10. The PU content was fixed at 10 wt%, ensuring that any differences in performance between P10 and DP10 could be attributed to the presence of disulfide-containing chain segments rather than to differences in total PU content.

#### 2.2.3. SBS-Modified Binders (S3 and S10)

SBS-modified binders were produced by mechanically blending SBS pellets into the base asphalt. The asphalt was heated to approximately 170 °C and stirred to a fluid state. SBS was gradually added to reach polymer contents of 3 wt% and 10 wt% for S3 and S10, respectively.

High-shear mixing was applied at about 6000 rpm for 30 min to ensure good dispersion of SBS within the asphalt matrix. The mixing temperature and time were controlled to minimize SBS degradation while enhancing SBS dispersion and possible polymer-rich phase development. After mixing, the binders were kept at elevated temperature for a short period to allow relaxation and removal of entrapped air before further testing.

### 2.3. Fourier Transform Infrared Spectroscopy (FTIR)

FTIR was used to identify functional groups and to confirm the formation of polyurethane structures and the incorporation of disulfide-containing segments in the modified binders. Thin films of each binder (BA, P10, DP10, S3, and S10) were prepared by casting a small amount of hot binder onto plates and allowing it to cool to room temperature.

Spectra were recorded in the wavenumber range of 600–4000 cm^−1^ with a resolution of 4 cm^−1^, using a Bruker TENSOR 27 FTIR spectrometer (Markham, York, ON, Canada) equipped with an Attenuated Total Reflection (ATR) geometry. Key absorption bands associated with N–H stretching, carbonyl stretching of urethane and ester groups, C–O–C stretching, and C–S stretching were analyzed and compared across the different binders.

### 2.4. Rheological Tests

All rheological tests were carried out using a Malvern Kinexus Dynamic Shear Rheometer (DSR) (Malvern, Worcestershire, UK) equipped with parallel plate geometries. Before testing, binders were conditioned according to the same thermal history to ensure comparability.

#### 2.4.1. High-Temperature Performance Grading

The high-temperature rutting resistance of the binders was evaluated according to relevant standards (ASTM D7643). A 25 mm plate with a 1 mm gap was used. Tests were performed in strain-controlled mode with an applied shear strain of 12% and an angular frequency of 10 rad/s. The complex modulus $G^{*}$ and phase angle δ were measured over a temperature range (e.g., 46–76 °C with 6 °C increments). The rutting parameter $G^{*}/\sin\delta$ was calculated at each temperature, and the high-temperature PG was determined based on the standard failure criterion. The results provide a first comparison of the high-temperature stiffness of BA, P10, DP10, S3, and S10.

#### 2.4.2. Time Sweep Fatigue Tests

Time sweep tests were used to evaluate the evolution of stiffness and damage under constant-strain cyclic loading. Tests were conducted at 25 °C using an 8 mm parallel plate geometry with a 2 mm gap. A sinusoidal shear strain with a fixed amplitude (e.g., 5%) and an appropriate loading frequency (e.g., 10 Hz) was applied until significant stiffness reduction occurred. The complex modulus $G^{*}$ was recorded as a function of the number of loading cycles. The number of cycles corresponding to a defined reduction in modulus (e.g., 20% decrease in $G^{*}$) was used as a fatigue failure criterion. In addition, the dissipated energy per cycle and the dissipated energy ratio (DER) were calculated to describe damage accumulation and to provide an alternative fatigue failure index.

#### 2.4.3. Linear Amplitude Sweep (LAS) Tests

The fatigue resistance of the binders over a range of strain amplitudes was further evaluated using the Linear Amplitude Sweep (LAS) test following AASHTO TP 101. Tests were carried out at 25 °C with an 8 mm plate and 2 mm gap. The LAS protocol consisted of two stages. First, a short frequency sweep at small strain was performed to obtain the linear viscoelastic properties of the undamaged binder. Second, a strain-controlled amplitude sweep was applied at a constant loading frequency (10 Hz), where the strain amplitude was increased linearly from a small value (e.g., 0.1%) to a large value (30%) over a fixed number of cycles (310 cycles).

The recorded shear stress and modulus were analyzed using a simplified viscoelastic continuum damage (VECD) approach to relate fatigue life $N_{f}$ to maximum strain $\gamma_{max}$ through a power-law relationship:

(1) $$N_{f}=A{\gamma_{max}}^{B},$$

where A and B are curve-fitting parameters that characterize the fatigue resistance at a reference strain and the sensitivity of fatigue life to strain amplitude, respectively. These parameters were obtained for BA, P10, DP10, and SBS-modified binders and are later summarized in a table for comparison.

#### 2.4.4. Fatigue–Healing–Fatigue Protocol

Healing capability was assessed using a fatigue–healing–fatigue (F–H–F) protocol in the DSR. An 8 mm plate with 2 mm gap was used at an intermediate temperature of 25 °C for fatigue loading and a slightly elevated temperature (i.e., 40 °C) for healing. Each test consisted of three stages:

1. First fatigue loading: the binder was subjected to strain-controlled cyclic loading (same conditions as in the time sweep test) until its complex modulus decreased to a predetermined fraction of the initial value (i.e., 60% of the original $G^{*}$). The modulus at the end of this stage is denoted $G_{0}^{\prime }$
2. Healing stage: the specimen was unloaded and kept at the healing temperature (i.e., 40 °C) for a fixed time (i.e., 5 min) without load. After healing, the complex modulus was re-measured and denoted $G_{1}$
3. Second fatigue loading: the binder was subjected again to cyclic loading under the same conditions as in the first stage. The evolution of modulus and damage was monitored to evaluate residual healing capability.

The healing index (HI) was defined as:

(2) $$HI=\frac{G_{1}-G_{0}^{\prime }}{{G_{0}-G}_{0}^{\prime }}\times 100\%,$$

where $G_{0}$ is the initial modulus before the first fatigue stage, $G_{0}^{\prime }$ is the modulus after the first fatigue stage, and $G_{1}$ is the modulus after healing. HI was calculated for the first healing cycle (${HI}_{1}$) and, where applicable, for a second healing cycle (${HI}_{2}$) to assess the ability of the binders to sustain repeated healing.

## 3. Results and Discussion

### 3.1. High-Temperature Performance

The high-temperature properties and PG of the binders are summarized in Table 3. Compared with the base asphalt, both PU-modified binders (P10 and DP10) and SBS-modified binders (S3 and S10) exhibited higher softening points and complex modulus values at the design high temperature, indicating improved high-temperature rutting resistance.

Both PU-modified binders showed a pronounced increase in $G^{*}/\sin\delta$ compared with BA, reflecting the formation of a stiff three-dimensional network. The presence of dynamic disulfide bonds in DP10 did not significantly reduce high-temperature stiffness relative to P10, suggesting that the introduction of dynamic bonds can preserve rutting resistance while potentially improving fatigue and healing performance. SBS-modified binders also increased high-temperature stiffness, with S10 showing the largest improvement due to the higher polymer content.

### 3.2. FTIR Analysis

**Figure 2:**
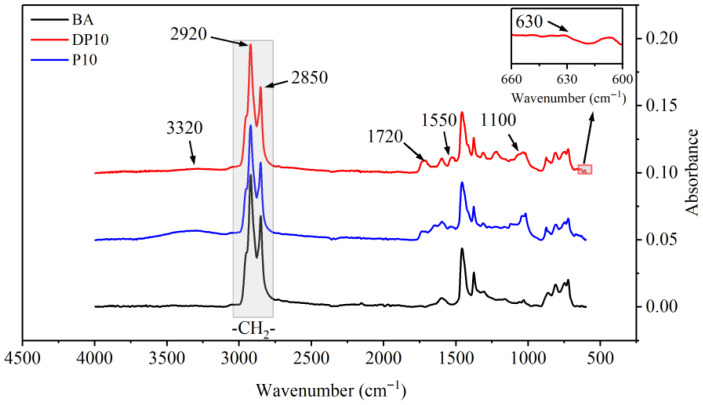
FTIR test results of asphalt binders. Note: Since the FTIR spectra are vertically translated for clarity, the ordinate values are in arbitrary units.

Figure 2 compares the FTIR spectra of BA, P10, and DP10. The base asphalt spectrum mainly shows bands associated with aliphatic C–H stretching (around 2920 and 2850 cm^−1^), C–H bending, and aromatic C=C stretching characteristic of bitumen.

For P10 and DP10, new absorption bands appear that are typical of polyurethane structures. A broad band near 3320 cm^−1^ corresponds to N–H stretching vibrations, while a band around 1550 cm^−1^ can be assigned to N–H bending. A band near 1720 cm^−1^ arises from C=O stretching in urethane and ester groups, indicating formation of urethane linkages through reactions between isocyanate and hydroxyl groups. Peaks in the region of 1100 cm^−1^ are associated with C–O–C stretching in the polyether backbone.

Importantly, DP10 exhibits an additional band in the low wavenumber region (around 630 cm^−1^) that is not observed in BA or P10. This band is attributed to C–S stretching in disulfide-containing moieties, confirming that the HEDS chain extender was successfully incorporated into the PU network within the asphalt binder. The FTIR results therefore demonstrate that (1) both PU-modified binders contain a chemically bonded polyurethane phase and (2) disulfide-containing segments are incorporated specifically in the DP10 network, providing a chemical basis for the different fatigue and healing behaviors observed in subsequent tests. It should be noted that FTIR provides chemical evidence of sulfur-containing segment incorporation, but it does not directly quantify disulfide exchange kinetics; thus, the proposed dynamic-network mechanism is inferred from the repeatable healing response rather than directly verified at the molecular level.

### 3.3. Time Sweep Fatigue Behavior

**Figure 3:**
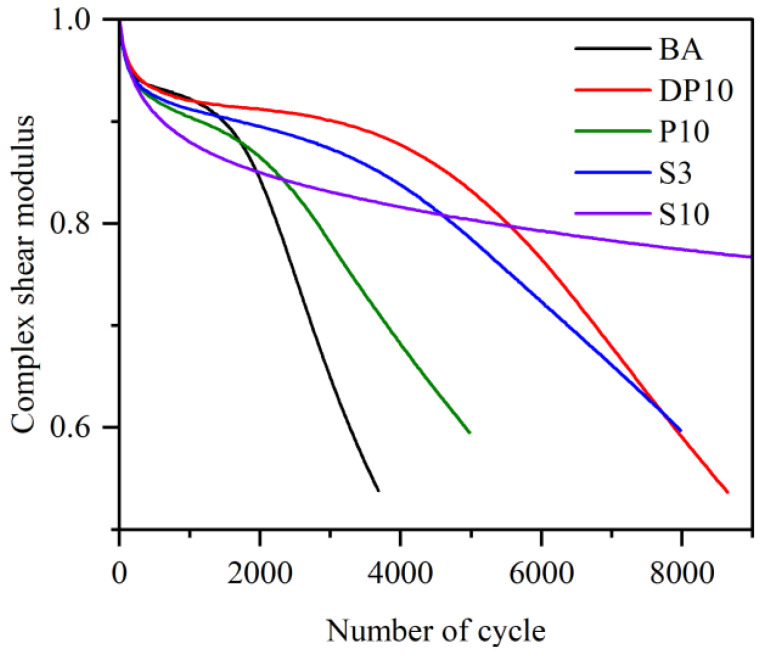
Complex shear modulus vs. number of cycles in terms of different asphalt binders.

The evolution of complex modulus $G^{*}$ as a function of loading cycles from the time sweep tests is shown in Figure 3. The base asphalt (BA) displayed the lowest initial modulus and the shortest plateau region of nearly constant stiffness. After a relatively small number of cycles (on the order of 10^3^), the modulus of BA started to decrease markedly, and a 20% reduction in $G^{*}$ was reached after approximately 2.3 × 10^3^ cycles, indicating limited fatigue resistance at the applied strain level.

Incorporating thermosetting PU (P10) significantly increased the initial modulus and extended the plateau region. The number of cycles required to reach the 20% modulus reduction criterion increased to roughly 3.0 × 10^3^ cycles, demonstrating that the rigid PU network can better withstand cyclic deformation before noticeable stiffness loss occurs. However, once damage initiated, the modulus of P10 decreased relatively rapidly, suggesting that microcracks in the brittle network are difficult to arrest or heal.

The disulfide-crosslinked binder DP10 showed both a high initial modulus and the longest plateau region among the PU-modified binders. The number of cycles to 20% modulus reduction increased to around 5.5 × 10^3^ cycles, representing a substantial improvement compared with both BA and P10. This indicates that the introduction of dynamic disulfide bonds into the PU network delays damage initiation and slows down stiffness degradation under constant-strain cyclic loading. By comparison, the SBS-modified binders displayed intermediate behavior: they improved initial modulus and fatigue life relative to BA, but their plateau length and number of cycles to failure were generally lower than those of DP10 at the same strain level.

**Figure 4:**
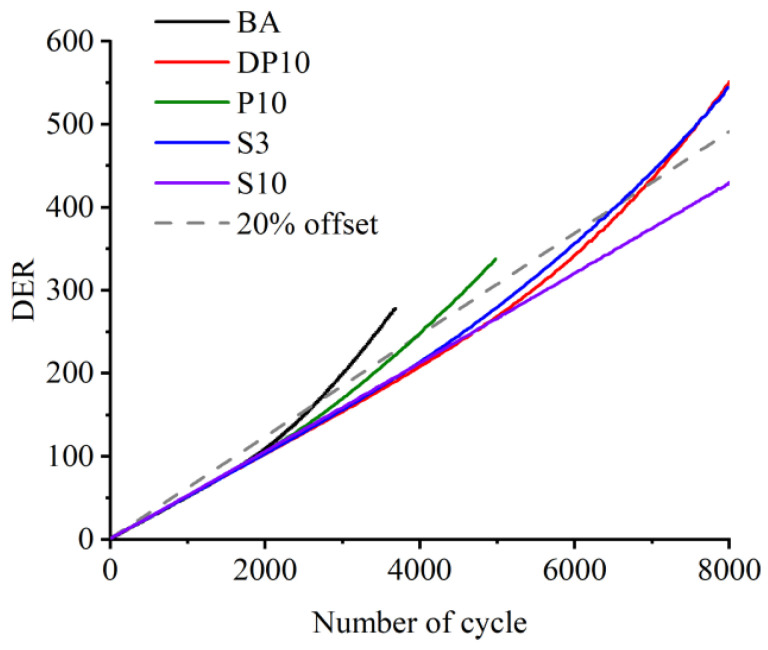
Dissipated energy ratio (DER) vs. number of cycles in terms of different asphalt binders.

The dissipated energy ratio (DER) curves plotted against the number of cycles provide additional insight into damage evolution, as presented in Figure 4. For all binders, a gradual increase in DER with small fluctuations was found initially, and DER then increased sharply as damage accumulated. The number of cycles at which DER deviated from its initial value by a set threshold was used as an alternative fatigue failure criterion. The ranking of binders based on DER was consistent with that based on modulus reduction: DP10 exhibited the highest fatigue life, followed by P10 and BA. The DER curves of SBS-modified binders intersected that of DP10, indicating that SBS systems perform well at later stages of fatigue, whereas DP10 is particularly effective in postponing early damage. Overall, the time sweep results demonstrate that dynamic disulfide-crosslinked PU can substantially enhance fatigue resistance compared with base asphalt and conventional thermosetting PU, providing both high stiffness and delayed damage onset.

### 3.4. LAS-Based Fatigue Performance

The LAS tests were used to quantify fatigue life over a wide range of strain amplitudes. The fitted fatigue parameters A and B for BA, P10, DP10, and S3 are listed in Table 4. Briefly, A reflects the fatigue resistance level at a reference strain (intercept-like scaling), while B indicates the strain sensitivity (damage acceleration) where a more negative B implies stronger reduction of $N_{f}$ with increasing strain. The base asphalt had the lowest A value, confirming its limited fatigue resistance at a reference strain level. Both PU-modified and SBS-modified binders showed much higher A values, indicating improved tolerance to cyclic loading. Among them, DP10 had the largest A, reflecting the best fatigue performance at small strain amplitudes.

The exponents B were negative for all binders, as expected. The magnitudes of B for P10 and DP10 were slightly larger than for BA, implying that their fatigue life is more sensitive to strain amplitude. However, the strain-sensitivity exponent B of DP10 was significantly less negative than that of P10 (two-sided *t*-test, *p* < 0.05, N= 3), indicating that the disulfide-containing PU network reduces the rate at which fatigue life decreases with increasing strain amplitude. The SBS-modified binder S3 showed an intermediate behavior with respect to B.

**Figure 5:**
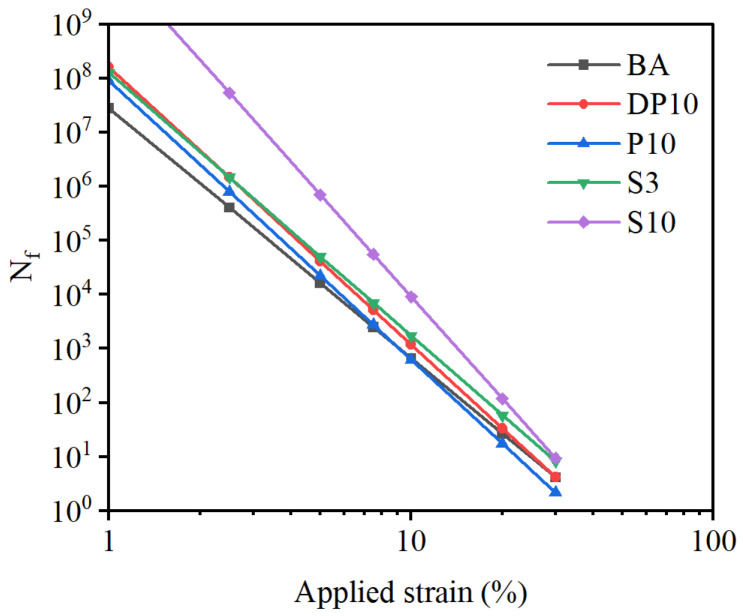
Calculated fatigue life vs. applied strain level in terms of different asphalt binders.

The predicted fatigue life curves $N_{f}$ in Figure 5 highlight these trends. At low strain levels (e.g., below about 2.5%), DP10 exhibits the longest predicted fatigue life among all binders, clearly outperforming P10 and BA and slightly surpassing S3. As the strain amplitude increases, the fatigue lives of all binders decrease, but the decrease is particularly steep for the thermosetting PU binder P10, which can even fall below that of BA at very high strain levels. This behavior reflects the brittleness and limited deformability of the rigid PU network once its elastic limit is exceeded.

By contrast, DP10 maintains a more favorable fatigue–strain relationship across the entire strain range. Although its fatigue life also decreases with increasing strain, it remains higher than that of P10 and BA and competitive with that of SBS-modified binders. These results confirm that dynamic disulfide crosslinks help the PU network to accommodate larger strains before catastrophic damage occurs, thereby extending the effective fatigue performance envelope of the binder.

### 3.5. Fatigue–Healing–Fatigue Behavior and Healing Index

**Figure 6:**
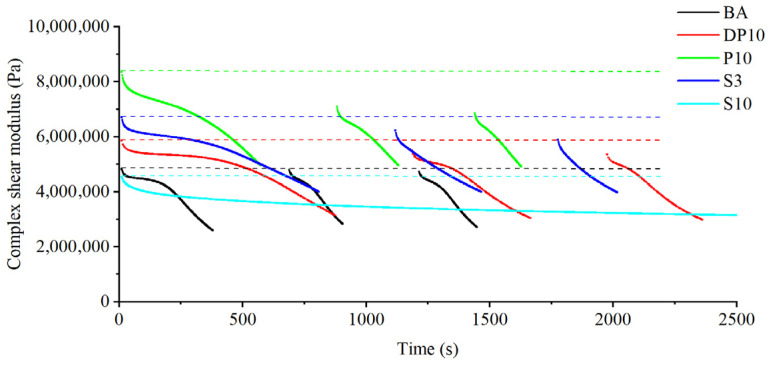
Complex shear modulus vs. test time from fatigue-healing-fatigue test in terms of different asphalt binders.

The fatigue–healing–fatigue tests provide direct information on the ability of the binders to recover stiffness after damage. Figure 6 presents a typical evolution of complex modulus during the repeated fatigue–healing–fatigue cycles (i.e., first fatigue loading stage, the healing stage, and the second fatigue loading stage) for the different binders. The healing indices for cycle 1 and cycle 2 are evaluated independently for each cycle.

The base asphalt showed a rapid modulus reduction under cyclic loading, consistent with its low initial stiffness, but recovered almost all of its original modulus after the healing period at 40 °C. This behavior is reflected in a healing index HI close to 100% for both the first and second healing cycles, confirming the strong intrinsic healing capability of bitumen.

The thermosetting PU binder P10 exhibited the highest initial modulus and resisted modulus reduction during the early part of the loading stage. However, after significant damage accumulated, its modulus dropped sharply. The modulus recovery after healing was limited, leading to the lowest HI among all binders in the first healing cycle. This indicates that the crosslinked PU network in P10 has very restricted ability to reform bonds or rearrange after damage, so most of the recovery arises from the asphalt matrix rather than from the PU phase. In the second cycle, the healing index of P10 improved slightly but remained lower than those of BA and other modified binders, reflecting residual damage in the network.

SBS-modified binders with normal dosage of 3% exhibited intermediate behavior. Their initial modulus and fatigue resistance were better than those of BA, and their healing indices were moderately high, benefiting from both the viscoelastic recovery of the asphalt matrix and the elasticity of the SBS-rich phase. However, the healing indices of SBS binders generally remained lower than those of BA, indicating that SBS does not fully preserve the intrinsic healing capability of the base binder.

**Figure 7:**
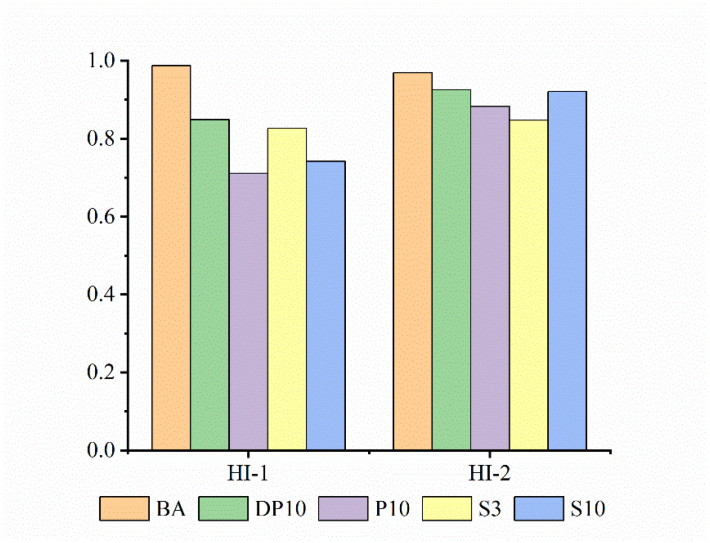
Self-healing parameters at first healing stage (${HI}_{1}$) and second healing stage (${HI}_{2}$) in terms of different asphalt binders.

The calculated healing indices including ${HI}_{1}$ and ${HI}_{2}$ are summarized in Figure 7. Although the exact values depend on the specific test conditions and failure levels, the trends are clear: P10 shows the poorest recovery, SBS binders show moderate recovery, and DP10 nearly restores the healing capacity of the base asphalt while maintaining higher stiffness and fatigue resistance. These results demonstrate that dynamic disulfide bonds in the PU backbone provide a mechanism for network rearrangement and bond exchange under moderate thermal conditions, enabling repeated healing of the binder after fatigue damage.

In particular, the disulfide-crosslinked binder DP10 combined desirable aspects of both systems. During the first fatigue loading stage, DP10 delayed modulus reduction and exhibited a longer plateau region than BA and P10, consistent with the time sweep results. After the healing period, DP10 recovered a large fraction of its initial modulus, yielding a healing index ${HI}_{1}$ much higher than that of P10 and comparable to that of SBS-modified binders. In the second fatigue–healing cycle, DP10 maintained high healing indices, approaching the values of the base asphalt.

### 3.6. Mechanistic Interpretation of Dynamic Disulfide Networks

The combined FTIR, rheological, and healing results allow a mechanistic interpretation of how dynamic disulfide bonds modify the behavior of PU-modified asphalt binders. In thermosetting P10, the polyurethane network is built predominantly from irreversible covalent bonds between polyol and isocyanate segments. This network architecture provides high stiffness and improved high-temperature performance, but limits chain mobility and prevents significant bond reformation once microcracks appear. As a result, P10 shows strong early fatigue resistance but poor recoverability and steep fatigue life decay at high strains.

In DP10, a portion of the network is constructed using disulfide-containing chain extenders. The disulfide bonds can undergo reversible exchange reactions under the moderate temperatures used in the healing stages. This dynamic covalent chemistry introduces additional degrees of freedom into the PU network: chain segments can temporarily detach and reattach, allowing local stress relaxation, microcrack closure, and partial reformation of the network after loading.

At small to medium strain levels, this increased mobility enables DP10 to better redistribute stress and delay the accumulation of irreversible damage, which explains its longer plateau region and higher fatigue life in the time sweep and LAS tests compared with P10 and BA. During rest periods at slightly elevated temperature, disulfide exchange reactions and segmental motion facilitate stiffness recovery, leading to high healing indices over repeated fatigue–healing cycles.

SBS-modified binders improve fatigue performance mainly by introducing an elastomeric phase that can store and release mechanical energy. However, this phase is not designed for bond exchange and does not intrinsically heal in the same way as dynamic covalent networks. Consequently, SBS binders show good fatigue resistance but moderate healing capability.

In sum, the results indicate that dynamic disulfide-crosslinked PU networks offer a promising strategy to reconcile the traditional trade-off between strength and healing in polymer modified asphalt binders. By tuning the fraction of dynamic bonds and the network architecture, it should be possible to design binders that combine high stiffness and rutting resistance with robust fatigue life and intrinsic self-healing capability, providing a material basis for long-life, self-healing pavement structures. It should be noted that the observed balance between stiffness, fatigue resistance, and healing may depend on PU content and disulfide density, and that future work will systematically investigate a broader PU range (e.g., 5–15 wt%) and optimized HEDS substitution ratios.

## 4. Conclusions

This study explored a dynamic disulfide-crosslinked polyurethane-modified asphalt binder (DP10) to combine the high strength of thermosetting polyurethane systems with the recoverability required for long-life pavements. A base asphalt (BA), a thermosetting PU-modified binder without dynamic bonds (P10), and two SBS-modified binders (S3 and S10) were used as reference materials. FTIR results confirmed the successful formation of urethane structures in both PU-modified binders and identified characteristic absorption bands associated with disulfide-containing moieties in DP10, indicating that dynamic covalent bonds were integrated into the binder network.

Time sweep fatigue tests revealed that incorporating thermosetting PU into the binder markedly increased stiffness and delayed the onset of modulus reduction relative to the base asphalt. However, the purely thermosetting PU system (P10) exhibited limited resistance to cumulative damage once microcracks formed, and its fatigue life decreased sharply at large strain amplitudes. In contrast, the disulfide-crosslinked binder DP10 extended the plateau region of constant modulus and significantly increased the number of cycles to a given damage level. LAS analysis further showed that DP10 had a larger fatigue parameter A and a more favorable exponent B, reflecting higher fatigue resistance at small to medium strain levels and a lower sensitivity of fatigue life to changes in strain amplitude compared with BA and P10. In the low-strain regime, DP10 provided fatigue performance comparable to, or even exceeding, that of SBS-modified binders.

Healing behavior, assessed through fatigue–healing–fatigue protocols, highlighted a clear difference between thermosetting and dynamic PU networks. The thermosetting PU binder P10 showed the lowest healing index, confirming that its crosslinked network could not effectively recover stiffness after damage. The base asphalt exhibited the highest intrinsic healing, with healing indices close to 100% in repeated cycles. Notably, DP10 achieved healing indices much higher than those of P10 and close to the level of the base binder, and it maintained strong healing capability over multiple damage–healing cycles. These results indicate that dynamic disulfide bonds in the PU network provide additional chain mobility and enable bond exchange, which supports crack closure and partial reconstruction of the network after loading.

The overall comparison suggests that DP10 offers a more favorable balance between stiffness, fatigue resistance, and healing than both base asphalt and non-dynamic PU systems. While the thermosetting PU binder can significantly enhance early mechanical performance, its lack of recoverability may limit its long-term benefit under repeated loading. By contrast, the disulfide-crosslinked polyurethane network in DP10 allows the binder to withstand cyclic loading more effectively and to restore a substantial portion of its stiffness after rest periods. These findings demonstrate that dynamic covalent chemistry, and disulfide bonds in particular, provide a viable route to self-healing polyurethane-modified asphalt binders. Future work may extend this concept to mixture-level performance, optimize disulfide bond density, and investigate the combined effects of thermal aging and moisture on the durability of such dynamic PU-modified systems.

In summary, DP10 achieves (1) high-temperature stiffness comparable to thermosetting PU and high-dosage SBS, (2) improved fatigue resistance across strain levels relative to P10 and competitive with SBS, and (3) substantially enhanced and repeatable healing compared to P10, approaching the intrinsic healing of base asphalt.

## Figures and Tables

**Table 1:** Basic properties of the base asphalt used.

Properties	Test Result	Test Standard
Rutting factor at 64 °C (kPa)	1.08	ASTM D7643 [26]
Performance grade (PG)	PG 64-22	ASTM D7643
Softening point (10 °C)	50.8	ASTM D36 [27]
Penetration (25 °C, 0.1 mm)	78.0	ASTM D946 [28]

**Table 2:** Binder designations, descriptions, and compositions.

Binder	Description	Modifier Content (wt%)
BA	Base asphalt	0
P10	Thermosetting PU-modified asphalt	10% PU (PPG + MDI)
DP10	Disulfide-crosslinked PU-modified asphalt	10% PU (PPG + MDI + HEDS)
S3	SBS-modified asphalt	3% SBS
S10	SBS-modified asphalt	10% SBS

**Table 3:** High-temperature rheological properties of asphalt binders.

Binder	$G^{*}/\sin\delta$ at 64 °C	High-Temperature PG
BA	1.08	PG 64-xx
P10	3.80	PG 76-xx
DP10	3.61	PG 76-xx
S3	3.43	PG 76-xx
S10	7.60	PG 88-

**Table 4:** LAS parameters of asphalt binders (mean ± SD, N = 3).

Binder	A (Mean ± SD)	B (Mean ± SD)
BA	2.81 × 10^7^ ± 3.0 × 10^6^	−4.6267 ± 0.06
DP10	1.60 × 10^8^ ± 2.0 × 10^7^	−5.1348 ± 0.05
P10	9.02 × 10^7^ ± 1.2 × 10^7^	−5.1591 ± 0.05
S3	1.28 × 10^8^ ± 1.5 × 10^7^	−4.8703 ± 0.06
S10	1.66 × 10^10^ ± 2.0 × 10^9^	−6.2612 ± 0.08

## Data Availability

The original contributions presented in this study are included in the article. Further inquiries can be directed to the corresponding author.

## References

[1] Chen Z., Wang T., Pei J., Amirkhanian S., Xiao F., Ye Q., Fan Z. (2019). Low temperature and fatigue characteristics of treated crumb rubber modified asphalt after a long term aging procedure. J. Clean. Prod..

[2] Ghavibazoo A., Abdelrahman M., Ragab M. (2013). Effect of Crumb Rubber Modifier Dissolution on Storage Stability of Crumb Rubber–Modified Asphalt. Transp. Res. Rec. J. Transp. Res. Board.

[3] Cong P., Liu C., Han Z., Zhao Y. (2023). A comprehensive review on polyurethane modified asphalt: Mechanism, characterization and prospect. J. Road Eng..

[4] Goyal M., Agarwal S.N., Bhatnagar N. (2022). A review on self-healing polymers for applications in spacecraft and construction of roads. J. Appl. Polym. Sci..

[5] Chang K., Jia H., Gu S.-Y. (2019). A transparent, highly stretchable, self-healing polyurethane based on disulfide bonds. Eur. Polym. J..

[6] Wei K., Wu Y., Cao X., Yang X., Tang B., Shan B. (2023). Dual dynamic bonds approach for polyurethane recycling and self-healing of emulsified asphalt. Sci. Total. Environ..

[7] Lyu L., Li D., Chen Y., Tian Y., Pei J. (2021). Dynamic chemistry based self-healing of asphalt modified by diselenide-crosslinked polyurethane elastomer. Constr. Build. Mater..

[8] You G., Li X., Ren K., Ai T., Niu Y. (2025). Effect of Disulfide Bond Density on the Properties of Polyurethane/Epoxy Interpenetrating Networks. Materials.

[9] Lu P., Huang S., Shen Y., Zhou C., Shao L. (2021). Mechanical performance analysis of polyurethane-modified asphalt using molecular dynamics method. Polym. Eng. Sci..

[10] Wang C., Huang S., Chen Q., Ji X., Duan K. (2023). Materials, preparation, performances and mechanism of polyurethane modified asphalt and its mixture: A systematic review. J. Road Eng..

[11] Zhuang W., Ding T., Pang C., Jiao X., Geng L., Sun M. (2025). Mechanical Properties and Modification Mechanism of Thermosetting Polyurethane-Modified Asphalt. Coatings.

[12] Wang X.-Y., Sun Q., Wang S., Shao R.-Y., Su J.-F. (2023). Multiscale Mathematical Analysis of Influencing Factors and Experimental Verification of Microcrack Self-Healing Efficiency of Bitumen Composites Using Microcapsules. Materials.

[13] Jia M., Zhang Z., Yang N., Qi B., Wang W., Huang Z., Sun J., Luo F., Huang T. (2022). Performance Evaluation of Thermosetting and Thermoplastic Polyurethane Asphalt Mixtures. J. Mater. Civ. Eng..

[14] Hao Z., Shan B., Liu P., Wu Y., Cao X. (2024). Preparation and Characterization of a Novel Self-Healing Polyurethane-Modified Asphalt Based on Dynamic Disulfide Bond. J. Mater. Civ. Eng..

[15] Zhu Y., Deng H., Luo H., Luo Y., Chen Y., Chen Z., Zhang C. (2025). Progress in the development of self-healing polyurethane materials. Resour. Chem. Mater..

[16] Yang W., Wu M., Xu T., Deng M. (2023). Recent Progress in the Field of Intrinsic Self-Healing Elastomers. Polymers.

[17] Guo X., Liu F., Lv M., Chen F., Gao F., Xiong Z., Chen X., Shen L., Lin F., Gao X. (2022). Self-Healable Covalently Adaptable Networks Based on Disulfide Exchange. Polymers.

[18] Xu X., Yuan L., Cong P., Wang Z., Zhou X., Wang J., Liu J. (2024). Self-healing microcapsule properties improvement technology: Key challenges and solutions for application in asphalt materials. Constr. Build. Mater..

[19] Li X., Yu R., He Y., Zhang Y., Yang X., Zhao X., Huang W. (2019). Self-Healing Polyurethane Elastomers Based on a Disulfide Bond by Digital Light Processing 3D Printing. ACS Macro Lett..

[20] Jian X., Hu Y., Zhou W., Xiao L. (2017). Self-healing polyurethane based on disulfide bond and hydrogen bond. Polym. Adv. Technol..

[21] Yang F., Cong L., Li Z., Yuan J., Guo G., Tan L. (2022). Study on preparation and performance of a thermosetting polyurethane modified asphalt binder for bridge deck pavements. Constr. Build. Mater..

[22] Li G., Wang M., Yan K., Song X. (2025). Study on the Self-Healing Performance of Polyurethane/Graphene Oxide-Modified Asphalt Based on Dynamic Disulfide Bonds. Materials.

[23] Cong L., Yang F., Guo G., Ren M., Shi J., Tan L. (2019). The use of polyurethane for asphalt pavement engineering applications: A state-of-the-art review. Constr. Build. Mater..

[24] Sun M., Zheng M., Qu G., Yuan K., Bi Y., Wang J. (2018). Performance of polyurethane modified asphalt and its mixtures. Constr. Build. Mater..

[25] Li Z., Yang F., Yuan J., Cong L., Yu M. (2021). Study on preparation and pavement performance of polyurethane modified asphalt based on in-situ synthesis method. Constr. Build. Mater..

[26] (2022). Standard Practice for Determining the Continuous Grading Temperatures and Continuous Grades for PG Graded Asphalt Binders.

[27] (2006). Standard Test Method for Softening Point of Bitumen (Ring-and-Ball Apparatus).

[28] (2009). Standard Specification for Penetration-Graded Asphalt Cement for Use in Pavement Construction.

